# A replicator model with transport dynamics on networks for species evolution

**DOI:** 10.1007/s00285-025-02279-w

**Published:** 2025-09-12

**Authors:** A. Coclite, S. F. Pellegrino, T. Politi, M. Popolizio

**Affiliations:** Dipartimento di Ingegneria Elettrica e dell’Informazione (DEI), Politecnico di Bari, Via E. Orabona 4, 70125 Bari, Italy

**Keywords:** Replicator Dynamics, Network, Numerical Simulation, Nash Equilibria, 37C75, 37N25, 65L05, 91A22, 92C42

## Abstract

This paper proposes a network-based framework to model and analyze the evolution and dynamics of a marine ecosystem. The model involves two different length scales: the evolution of species in local reserves and the exchange of species between reserves. At the inter-reserve level, species evolution is ruled by the replicator equation, while a transport function accounts for the transport at the network level. This multi-scale approach allows for capturing both local dynamics within individual reserves and the broader connectivity and interactions across the network. We study how equilibria are modified due to the exchange between connected nodes and prove that evolutionarily stable states are asymptotically stable if the velocity transfer $\nu$ is contained within a condition involving the maximum degree of the network. A fourth-order P-(EC)$^k$ formulation of the Gauss-Legendre Runge Kutta scheme is adopted. This numerical procedure is challenged against a suitable numerical experiment involving three species on a single node for validating the robustness of the scheme in terms of accuracy for a large observation time. Several numerical experiments are provided for characterizing the abilities and limitations of the model. Three prototypical networks are considered for the case of two- and three-agent games with both linear and nonlinear transport terms. Moreover, the ability of the proposed model to reproduce synchronization phenomena on networks is discussed. This approach has been demonstrated to have the potential to uncover insights into the stability, resilience, and long-term behavior of these ecosystems, offering valuable tools for their conservation and management.

## Introduction

Our work originates within the framework of a project focused on the mathematical modeling of biodiversity in the Mediterranean Sea. Therefore, our primary interest lies in the analysis of marine systems. However, the analysis presented here applies to a broad class of dynamic systems that extend beyond the specific context of marine environments. A crucial feature of marine ecosystems is that they are complex systems that evolve over multiple temporal and spatial scales, posing significant challenges for their study and management. To address these intricacies, we propose a network-based framework to model and analyze the dynamics of such a system.

Network theory has long been used to describe complex systems with interacting components. The nodes represent these components, which can be individual entities, communities, or cities, while the edges describe the interactions between the nodes. (Bertaglia and Pareschi 2021) were among the first to add an evolutionary process for the interactions between nodes. In this way, the spatial scale of the analysis, within individual nodes, was added to the temporal scale described by the interactions between nodes. Emerging applications are even more challenging, since they are characterized by large-scale collections of dynamical systems interacting with each other over a web of complex interconnections (D’Souza et al. 2023). A different approach was proposed in (Nurisso et al. 2024), where the authors studied the interaction of the multiagent system by deducing a system of kinetic equations on graphs. They showed that a statistical description of the graph topology can capture the aggregate trends of networked interacting systems.

In this framework, marine reserves are represented as the network nodes, while the edges describe the interactions between reserves, interpreted as the movement or dispersal of species between them. Each node itself represents a dynamic system characterized by the presence of multiple interacting species. The intra-reserve dynamics are governed by the replicator equation, a well-known model for evolutionary dynamics that captures species interactions and population dynamics, recently interpreted as the large-population limit of the Moran (Morandotti and Orlando 2025). At the inter-reserve level, we describe species movement using transport equations, which account for the exchange of populations between reserves over time. This multi-scale approach allows us to capture both local dynamics within individual reserves and the broader connectivity and interactions across the network. By integrating replicator dynamics at the node level with transport equations at the network level, this study aims to provide a comprehensive understanding of the evolution of marine systems. This approach has the potential to uncover insights into the stability, resilience, and long-term behavior of these ecosystems, offering valuable tools for their conservation and management. A delicate challenge lies in the magnitude of interactions that span intensity and spatio-temporal scales, from long-range maritime traffic to short-range commuting flows. The analysis of the spread of human infectious diseases shares the same difficulties, since human mobility represents a crucial point both on the theoretical side and given the limited availability of empirical data. Here, many models employ data-driven approaches for long-range mobility at the inter-population level, combined with coarse-grained techniques for dynamics within each sub-population (see (Balcan et al. 2009) and references therein).

The manuscript is organized as follows. In Section 1 we introduce the replicator model and recall the properties of equilibria depending on the assumptions on the involved payoff matrix. In Section 2, we extend the replicator dynamics to a network. We parametrize the dynamics among the nodes by a transport term and study how equilibria are modified due to the exchange between connected nodes. In Section 3, we propose a discretization of the model by the implicit fourth-order Gauss-Legendre Runge-Kutta method and validate its implementation in the case where the model is applied on a single node. Section 3.2 is devoted to numerical simulations on networks with different configurations. Finally, Section 4 concludes the paper.

## The replicator equation

The replicator equation was first introduced in 1978 by Taylor and Jonker in the context of game theory. Still, it is also widely used in population ecology and generally to describe evolutionary dynamics within a population. The literature on this topic is very rich and varied. We refer to the texts by (Nowak 2006) and Hofbauer and Sigmund (Sigmund and Hofbauer 1998) and references therein.

Assume that we deal with a population divided into *n* species with time-dependent frequencies $x_1(t)$ to $x_n(t)$. Clearly,

1.1 $$\begin{aligned} \sum _{k=1}^n x_k(t) =1, \text {for any} \,\, t>0. \end{aligned}$$

The evolution of these frequencies is related to a specific function, called *fitness*, which represents the individual’s ability to survive and subsequently reproduce. It is a function of the composition of the population and, as is usually done, we assume it to be a linear function, that is, there is a square matrix *A* of dimension $n\times n$, such that the fitness of the *k*-th species is expressed as $(Ax)_k$. In this context, *A* is called the *fitness matrix* and the replicator equation reads as follows:

1.2 $$\begin{aligned} \dot{x_k} = x_k ((Ax)_k - x^TAx) \end{aligned}$$

defined on the simplex

1.3 $$\begin{aligned} S_n=\left\{ p=(p_1,\ldots ,p_n)\in \mathbb {R}^n : {p_k} \ge 0 \,\,{\text {and}} \,\,{\sum _{k=1}^n p_k =1}\right\} . \end{aligned}$$

Notably, the interior of the simplex is invariant: if a trajectory begins inside it, it will always remain there. While it may approach the boundary, it will never actually reach it. A face of the simplex is a subset in which at least one strategy has a frequency of zero. Faces are invariant under (1.2).

The replicator equation represents the basic Darwinian principle that the growth rate of a phenotype in the population is given by the difference between the fitness of this phenotype and the average fitness of all phenotypes in the population. For large *n*, estimating the interaction matrix often becomes a daunting task. An alternative approach is to generate it randomly, characterizing its statistical distribution using a limited set of model parameters (May 1972; Akjouj et al. 2024).

An interior equilibrium of (1.2) is given by the solution of the linear system of equations $(Ax)_k=x^TAx$, in addition to (1.1) and $x_k>0$ for $k=1,\ldots ,n$. This system has one or zero nondegenerate solutions. Thus, there can be at most one isolated equilibrium in the interior of the simplex $S_n$. If there is no equilibrium in the interior, then all trajectories converge to the boundary of the simplex. Thus, there can be no chaotic attractor and no limit cycle in the interior if there is no equilibrium in the interior. This result is very helpful because sometimes it is possible to show that a particular replicator equation admits no interior equilibrium. If this is the case, then we know that nothing more complicated can happen in the interior; co-existence of all strategies is impossible. In degenerate cases, the replicator equation can admit a manifold of equilibria in the interior. These equilibria can be stable, but not asymptotically stable. In what follows, we characterize the equilibria for replicator models for two and three species.

### Replicator dynamics and the Lotka-Volterra equation

The Lotka-Volterra equations were developed from the works of Lotka (1925) and Volterra (1926) in separate publications to describe the evolution of a system with competing species. For *n* populations, the general Lotka-Volterra model reads:

1.4 $$\begin{aligned} {\dot{x}}_k=x_k\left( r_k+\sum _{j=1}^n a_{kj}x_j\right) ,\,\,\, k=1,\ldots ,n\, , \end{aligned}$$

where $x_k$ describes densities, $r_k$ are intrinsic growth (or decay) rates, and $a_{kj}$ describes the effect of species *j* on species *k*. Interestingly, the replicator equation in *n* variables is equivalent to the Lotka-Volterra equation in $n-1$ variables (Hofbauer 1998), so results about Lotka-Volterra equations can be carried over to the replicator equation and vice versa. However, the two model differ in the physical meaning of their evolving variables: the Lotka-Volterra equations describe the population sizes of interacting species (typically in predator-prey systems), whereas replicator dynamics tracks the relative frequencies of strategies in a population, which evolve over time according to their payoff relative to the average.

## The replicator model on networks

This paper analyzes the dynamics of *n* species coexisting in a biological ecosystem. The spatial dispersion of this ecosystem is modeled by a network consisting of *N* nodes connected by edges. Such networks are supplied with an adjacency matrix *M* defined as a square matrix where each entry $M_{ij}$ encodes the presence or absence of a direct connection between nodes *i* and *j*. Specifically, $M_{ij} = 1$ if there is an edge connecting *i* and *j*, and $M_{ij} = 0$ otherwise. As such, for all $i \in \{1, \dots , N\}$, the set *c*(*i*) is defined as the set of nodes adjacent to node *i*, namely $c(i) = \{ j \mid M_{ij} = 1 \}$. In the following, for clarity, the network nodes are parameterized by the index $i\in \mathcal {I}=\{1,\dots ,N\}$ and the species by the index $k\in \mathcal {K}=\{1,\dots ,n\}$. Let $\textbf{x}^i(t):\mathbb {R}^+\rightarrow [0,1]^n$ be the density of the node collecting the specific densities of the *n* species at the node *i* at a fixed time $t > 0$; so that $x^i_k(t)$ is the density of beings belonging to the *k*-th species and living in the *i*-th node. This nodal density function is such that the sum of all species within a node is unitary, so $\textbf{x}^i(t) \in S_n\,\, \forall t > 0 \text { and } i \in \mathcal {I}$.

Species may travel back and forth on an edge that connects two nodes. Let *i* and *j* be the indices of two connected nodes. The species transfer rate between the node *i* and all nodes connected to it $\textbf{T}(\textbf{x}^i(\cdot )): [0,1]^n\rightarrow [0,1]^n$ is chosen as a function of the variation in species densities between the selected nodes. In this work, two functional forms for such a rate are analyzed:

2.1 $$\begin{aligned}&\textbf{T}^{\nu }(\textbf{x}^i(\cdot ))(t) = \sum _{j\in c(i)} \nu _{i,j} \left( \textbf{x}^j(t) - \textbf{x}^i(t)\right) \, , \end{aligned}$$

2.2 $$\begin{aligned}&\textbf{T}^{p}(\textbf{x}^i(\cdot ))(t) = \sum _{j\in c(i)} e^{-p^{ij} \mathbf{\tau }^{ij}} \left( \textbf{x}^j(t) - \textbf{x}^i(t-\tau ^{ij})\right) \, , \end{aligned}$$

being *c*(*i*) the set of nodes connected to *i*; $\nu _{ij}$ a positive constant having the physical meaning of a transfer velocity; $p^{ij}$ the survival rate associated with the edge connecting *i* to *j*; and $\tau ^{ij}$ the time needed for a species to complete the journey. In particular, the two functional forms for the transfer rate are based on two different assumptions. $\textbf{T}^{\nu }(\textbf{x}^i(\cdot ))$ would represent the most simplifying rate returning species migrating with velocity $\nu _{ij}$ on a network in which all nodes may be differently separated; on the other side, $\textbf{T}^{p}(\textbf{x}^i(\cdot ))$ is modeled as exponentially decaying with $\tau ^{ij}$ and modulated by a mortality coefficient $p^{ij}$. The dynamics of the *k*-th species within a node is regulated by the replicator equation, returning the following system for the evolution of the entire ecosystem:

2.3 $$\begin{aligned} &  \dot{x}^i_k(t) = x^i_k(t) \left( \left( A^i \textbf{x}^i(t)\right) _k - (\textbf{x}^i(t))^TA^i \textbf{x}^i(t)\right) + T_k \left( \textbf{x}^i(\cdot )\right) (t)\, , \nonumber \\ &  \quad \text { for } k \in \mathcal {K}, \text { and }\, i\in \mathcal {I}, \end{aligned}$$

$A^i \in \mathbb {R}^{n\times n}$ being the payoff matrix regulating the coexistence of species within the node *i*.

### Stability analysis of the model with linear transport

In this section, we present the stability analysis of the model described by (2.3)-(2.1). For simplicity, we assume to have the same payoff matrix for all nodes, i.e. $A^i=A$ for any $i\in \mathcal {I}$, and the same transport coefficient on the whole network, say $\nu$. We begin by introducing the fundamental definitions of game theory relevant to the model described by the replicator equation, following (Sigmund and Hofbauer 1998). Building on these definitions, we analyze the stability of the model that integrates transport and replicator dynamics, emphasizing the role of the transport coefficient.

#### Definition 2.1

A state $x^*\in S_n$ is a *Nash equilibrium* if for all $x\in S_n$, $x\ne x^*$:

2.4 $$\begin{aligned} \left( x^*\right) ^T A x^*\ge x^T A x^*\, . \end{aligned}$$

#### Definition 2.2

A state $x^*\in S_n$ is an *evolutionarily stable state* if for all $x\in S_n$, $x\ne x^*$ and for all $\varepsilon >0$:

2.5 $$\begin{aligned} \left( x^*\right) ^T A \left( \varepsilon x +(1-\varepsilon ) x^*\right) > x^T A \left( \varepsilon x +(1-\varepsilon ) x^*\right) \, . \end{aligned}$$

#### Theorem 2.3

A state $x^*\in S_n$ is an evolutionarily stable state in the sense of Definition 2.2 if and only if for all $x\in S_n$, $x\ne x^*$ in a neighborhood of $x^*$:

2.6 $$\begin{aligned} \left( x^*\right) ^T A x > x^T A x \, . \end{aligned}$$

#### Definition 2.4

A fixed point of the replicator dynamics (or any dynamical system) is said to be *asymptotically stable* if any small deviations from that state are eliminated by the dynamics as $t\rightarrow \infty$.

The concepts of Nash equilibrium and evolutionarily stable state are related to the outcome of a game in which each individual in the population attempts to maximize its payoff. In this sense, a Nash equilibrium represents a state in which individuals would not gain benefits from changing their strategy. Instead, the concept of an evolutionary stable state can be explained in this way: if a population has chosen an evolutionary stable state $x^*$, any invader group with distribution close to $x^*$, cannot improve its average payoff. Thus, the invaders are extinguished. As a consequence, we can prove that evolutionarily stable states are asymptotically stable states if the velocity transfer $\nu$ is sufficiently small or the network is sufficiently sparse. For the following, we denote by $\left| c(i) \right|$ the number of nodes that are connected to the *i*-th node.

#### Theorem 2.5

Assume that the transfer between nodes is governed by $\textbf{T}^\nu \left( x\right)$ as in (2.1). Let $x^*$ be an evolutionarily stable state for each node. If the velocity transfer $\nu$ is small enough to guarantee that the following inequality holds

2.7 $$\begin{aligned} \left( x^*\right) ^T A x^i - \left( x^i\right) ^T A x^i - \nu \left| c(i)\right| > 0, \end{aligned}$$

for any $i\in \mathcal {I}$, then $x^*$ is asymptotically stable.

#### Proof

Let us define the following Lyapunov function,

2.8 $$\begin{aligned} V(x) = - \sum _{i\in \mathcal {I}}\sum _{k=1}^n x_k^{*} \ln {\left( \frac{x_k^i(t)}{x_k^{*}}\right) } \, . \end{aligned}$$

It is a positive definite function. Moreover, $V(x^{*})=0$ and the derivative along the trajectories of the system (2.3) is given by

$$\begin{aligned} {\dot{V}}(x) = -\sum _{i\in \mathcal {I}}\sum _{k=1}^n x_k^{*} \frac{{\dot{x}}_k^i(t)}{x_k^{i}}\, . \end{aligned}$$

Using equations (2.3) and (2.1), together with the conservation of density on each node and assumption (2.7), and noting that $x_k^i(t) \ge 0$ for all $i \in \mathcal {I}$, $k \in \mathcal {K}$, and $t > 0$, we obtain

$$\begin{aligned} \begin{aligned} {\dot{V}}(x) =&-\sum _{i\in \mathcal {I}}\sum _{k=1}^{n} x_k^{*}\left( \left( A x^i(t)\right) _k - x^i(t)^T A x^i(t) + \nu \sum _{j\in c(i)} \frac{x^j_k(t)-x^i_k(t)}{x_k^i(t)}\right) \\ =&-\sum _{i\in \mathcal {I}} \left( \left( x^*\right) ^T A x^i(t) -x^i(t)^T A x^i(t) - \nu \left| c(i)\right| +\nu \sum _{k=1}^{n}x_k^*\sum _{j\in c(i)}\frac{x_k^j(t)}{x_k^i(t)}\right) \, . \end{aligned} \end{aligned}$$

Since $x^*$ is an evolutionary stable state, ${\dot{V}}(x)$ is negative definite in a neighborhood of $x^*$. Thus, $x^*$ is asymptotically stable. $\square$

#### Remark 2.6

The above theorem states that if a strategy $x^*$ is evolutionarily stable at each node, then under the influence of a small enough velocity transfer $\nu$, it remains asymptotically stable. This implies that $x^*$ is attractive, which means that the trajectories that start nearby will converge to $x^*$ over time. Inequality (2.7) ensures that the stability condition is met even in the presence of transport effects. Moreover, assumption (2.7) can be interpreted as a constraint on $\nu$ that ensures the stability of the model, specifically:

$$ \nu <\frac{1}{\displaystyle \max _{i\in \mathcal {I}}\left| c(i)\right| } $$

where $\Delta =\displaystyle \max _{i\in \mathcal {I}}\left| c(i)\right|$ is usually defined as the *maximum degree* of the network.

The maximum degree $\Delta$ of a network provides partial information about its sparsity. In general, a low value of $\Delta$ suggests that the network is sparse.

Therefore, if the network is sufficiently sparse, assumption (2.7) holds and asymptotical stability is guaranteed.

A particular condition that guarantees that a Nash equilibrium is asymptotically stable is when the fitness matrix is diagonal with negative entries, as stated in the following result.

#### Theorem 2.7

Assume that the fitness matrix is diagonal with negative entries. Let $x^*$ be a Nash equilibrium satisfying the condition (2.7), then it is asymptotically stable under the replicator dynamics with transport given by (2.3)-(2.1).

#### Proof

Let $x^*$ be a Nash equilibrium. Thanks to Theorem 2.5, it is sufficient to prove that $x^*$ is an evolutionarily stable state. We fix $x\in S_n$, and we define the quantity

2.9 $$\begin{aligned} \psi (x) = \sum _{i=1}^n \left( x_i^*- x_i\right) \left( Ax\right) _i. \end{aligned}$$

Thus, adding and subtracting the quantity $\left( Ax^*\right) _i$ to equation (2.9) and using (2.4), we find

$$\begin{aligned} \begin{aligned} \psi (x)&= \sum _{i=1}^n \left( x_i^*- x_i\right) \left( Ax\right) _i = \sum _{i=1}^n \left( x_i^*- x_i\right) \left( \left( Ax\right) _i - \left( Ax^*\right) _i + \left( Ax^*\right) _i\right) \\&\ge \sum _{i=1}^n -a_{ii}\left( x_i^*- x_i\right) ^2. \end{aligned} \end{aligned}$$

Since $a_{ii}$ is strictly negative for every $i=1,\dots , n$, $\psi (x)$ is positive definite, and this concludes the proof. $\square$

The theorem above highlights that even in the presence of interactions between nodes, a well-structured system (namely, negative diagonal fitness values, Nash condition, and controlled transport) will stabilize around a Nash equilibrium. Let us give an interpretation to the situation described in the previous Theorem: assume that we have only two species. The fitness matrix in this case corresponds to:

$$ \begin{pmatrix} a^{\prime } & 0\\ 0 & b^{\prime } \end{pmatrix} $$

with $a^{\prime }, b^{\prime }$ negative. It is trivially verified that on $S_n$ the replicator equation (1.2) with this payoff matrix is equal to the replicator equation (1.2) with the payoff matrix

$$ \begin{pmatrix} a& b\\ c& d \end{pmatrix} $$

with $a^{\prime }=a-c$ and $b^{\prime }=d-b$. A diagonal payoff matrix with negative diagonal entries represents a situation where the players receive a negative payoff when they choose the same strategy, but there are no incentives to cooperate or coordinate. More specifically, such a matrix can be interpreted as a situation of conflict or competition where choosing the same strategy leads to losses for both players, or in the case of independent players. Notably, in this case, the system admits three Nash equilibria given by $(1,0), (0,1), \left( b^{\prime }/(a^{\prime }+b^{\prime }),a^{\prime }/(a^{\prime }+b^{\prime }\right) ).$ The last one is the unique asymptotically stable state for the model.

#### Remark 2.8

**Simplex Invariance:** Let $S_n$ denote the unit simplex defined in (1.3). Assume that $\textbf{x}^i$ for $i\in \mathcal {I}$ is a solution of (2.3) with transport as in (2.1) and $\nu$ such that (2.7) is verified. If $\textbf{x}^i(0)\in S_n$ then $\textbf{x}^i(t)\in S_n$ for any $t>0$.

#### Proof

We have to show that for $i\in \mathcal {I}$ and $t>0$, if $\sum _{k\in \mathcal {K}} x^i_k(0) = 1$ and $x_k(0)\ge 0$ for $k\in \mathcal {K}$, the following two propositions hold at the same time:

$$\begin{aligned} i)&\, \, \sum _{k\in \mathcal {K}} x^i_k(t) = 1\, ,\\ ii)&\, \, x^i_k(t) \ge 0 \ \ \text {for all}\, \, k\in \mathcal {K}\, . \end{aligned}$$

To prove *i*), we have to compute the time derivative of $\sum _{k\in \mathcal {K}} x^i_k(t)$:

2.10 $$\begin{aligned} &  d_t\left( \sum _{k\in \mathcal {K}} x^i_k(t)\right) = \sum _{k\in \mathcal {K}} d_t \left( x^i_k(t) \right) = \nonumber \\ &  = \sum _{k\in \mathcal {K}} \Bigl ( x^i_k(t) \left( \left( A^i \textbf{x}^i(t)\right) _k - (\textbf{x}^i(t))^TA^i \textbf{x}^i(t)\right) + T^\nu _k \left( \textbf{x}^i(\cdot )\right) (t) \Bigr ) = \nonumber \\ &  = \sum _{k\in \mathcal {K}} \left( x^i_k(t) \left( \left( A^i \textbf{x}^i(t)\right) _k - (\textbf{x}^i(t))^TA^i \textbf{x}^i(t)\right) + \nu \sum _{j\in c(i)} \left( x^j_k(t) - x^i_k(t)\right) \right) = \nonumber \\ &  = \underbrace{\sum _{k\in \mathcal {K}} \Bigl ( x^i_k(t) (\left( A^i \textbf{x}^i(t)\right) _k \Bigr )}_{F_1} - \underbrace{(\textbf{x}^i(t))^TA^i \textbf{x}^i(t)}_{F_2} \sum _{k\in \mathcal {K}} x^i_k(t) + \nu \sum _{j\in c(i)} \left( \sum _{k\in \mathcal {K}} \left( x^j_k(t) - x^i_k(t)\right) \right) \, . \nonumber \\ \end{aligned}$$

By observing that $F_1$ equals $F_2$ and due to the continuity of $\textbf{x}^i$, proposition *i*) follows from the assumption $\sum _{k\in \mathcal {K}} x^i_k(0) = 1$. Proposition *ii*) is strictly deduced from the continuity of $\textbf{x}^i$ and the Remark 2.6. In fact, since for all $k\in \mathcal {K}$ it must be $x^i_k(0) \in [0,1]$ and $\nu < \frac{1}{max_{i\in \mathcal {I}}|c(i)|}$, the continuity of $\textbf{x}^i(t)$ ensures that:

2.11 $$\begin{aligned} x^i_k(t) \left( \left( A^i \textbf{x}^i(t)\right) _k - (\textbf{x}^i(t))^TA^i \textbf{x}^i(t)\right) \ge \nu \sum _{j\in c(i)} \left( x^j_k(t) - x^i_k(t)\right) \, . \end{aligned}$$

The verification of propositions *i*) and *ii*) proves the simplex invariance. $\square$

## Numerical approximation and computational procedure.

For integrating (2.3), the selected numerical scheme needs to preserve the geometrical features of the solution for $t>0$:

$$\begin{aligned}&{\textbf {non-negativity of species:}} \qquad x^i_k(t) \ge 0 \, \, \text {for} \, \, k \in \mathcal {K}, \, \, \text {and} \, \, i \in \mathcal {I}; \\&{\textbf {unitary sum of species:}} \ \quad \qquad \sum _{k\in \mathcal {K}} x^i_k(t) = 1 \, \, \text {for} \, \, i \in \mathcal {I}\, . \end{aligned}$$

Moreover, it should be robust and highly accurate for a large observation time and handle nonlinearities efficiently. At this scope, the implicit fourth-order Gauss-Legendre Runge-Kutta scheme is adopted. It is a Runge-Kutta method suited for ordinary differential equations (ODEs) with high accuracy (Iserles 2009) based on the Gauss-Legendre quadrature nodes. As broadly recognized, a Gauss-Legendre method comprising *s* stages attains an order of accuracy 2*s*. Consequently, this enables the construction of methods with arbitrarily high order while maintaining a manageable computational effort. These methods are particularly notable for preserving important qualitative properties of the solution, such as symplectic structure and energy conservation, in Hamiltonian systems. Furthermore, such schemes are *A*-stable, providing an effective approach to handle stiff equations without the occurrence of numerical instability. Consequently, they are extensively adopted for integrating conservative systems, where sustaining long-term accuracy is essential. This method, applied to a general ODE $\dot{y(t)}=f(t,y)$ reads:

3.1 $$\begin{aligned} \begin{aligned}&k_1 = f\left( t_n + \Bigl (\frac{1}{2} -\frac{\sqrt{3}}{6}\Bigr )\Delta t\, , \, y_n + \Delta t\Bigl ( \frac{1}{4}k_1 + \Bigl (\frac{1}{4} -\frac{\sqrt{3}}{6}\Bigr )k_2 \Bigr ) \right) \\&k_2 = f\left( t_n + \Bigl (\frac{1}{2} + \frac{\sqrt{3}}{6}\Bigr )\Delta t\, , \, y_n + \Delta t\Bigl ( \Bigl (\frac{1}{4} + \frac{\sqrt{3}}{6}\Bigr )k_1 + \frac{1}{4}k_2 \Bigr ) \right) \\&y_{n+1} = y_n + \frac{1}{2}\Delta t ( k_1 + k_2) \end{aligned} \end{aligned}$$

where $$\Delta t$$ is the time step, $$k_1, k_2$$ are internal stages and $$ y_n$$ is the numerical solution at time level $$t_n$$. In this paper, we adopt a P-(EC)$$^k$$ formulation. Specifically, by rewriting Eq. (2.3) as:

3.2 $$\begin{aligned} {\dot{x}^i_k(t) = L_k\left( \textbf{x}^i(t)\right) }\, , \end{aligned}$$

with $\displaystyle L\left( \textbf{x}^i(t)\right) = x^i_k(t) \left( A^i \textbf{x}^i(t)\right) _k - (\textbf{x}^i(t))^TA^i \textbf{x}^i(t) + T_k \left( \textbf{x}^i(t)\right)$ and by denoting with $(\cdot )_n$ the n-th temporal level of a variable the numerical scheme reads:

- Explicit step for initial guesses:
  - $$\left( x_k^i\right) ^{P,1}_{n+1} = \left( x_k^i\right) _{n} + \left( \frac{1}{2}-\frac{\sqrt{3}}{6}\right) \Delta t L_k\left( \left( \textbf{x}^i\right) _{n}\right) $$,
  - $$\left( x_k^i\right) ^{P,2}_{n+1} = \left( x_k^i\right) _{n} + \left( \frac{1}{2}+\frac{\sqrt{3}}{6}\right) \Delta t L_k\left( \left( \textbf{x}^i\right) _{n}\right) $$;
  - $$k_1 = L_k\left( \left( x_k^i\right) ^{P,1}_{n+1}\right) $$,
  - $$k_2 = L_k\left( \left( x_k^i\right) ^{P,2}_{n+1}\right) $$;
  - $$k^C_1 = L_k\left( \left( \textbf{x}^i\right) _{n}+\frac{1}{4}k_1+ \left( \frac{1}{4}- \frac{\sqrt{3}}{6}\right) k_2 \Delta t\right) $$,
  - $$k^C_2 = L_k\left( \left( \textbf{x}^i\right) _{n}+ \left( \frac{1}{4}+ \frac{\sqrt{3}}{6}\right) k_1 + \frac{1}{4}k_2\Delta t \right) $$;
- Implicit steps (up to the criterion $max(\Vert k_1-k^C_1\Vert _{L^2},\Vert k_2-k^C_2\Vert _{L^2}) <\epsilon$ is reached) :
  - $$\begin{pmatrix} k_1\\ k_2\\ \end{pmatrix} = \begin{pmatrix} I-\Delta t\frac{1}{4}k^C_1 & -\Delta t\left( \frac{1}{4}- \frac{\sqrt{3}}{6}\right) k^C_1\\ -\Delta t\left( \frac{1}{4}+ \frac{\sqrt{3}}{6}\right) k^C_2 & I-\Delta t\frac{1}{4}k^C_2\\ \end{pmatrix} \begin{pmatrix} k_1-k^C_1 \\ k_2-k^C_2 \\ \end{pmatrix}$$;
  - Updating $$k^C_1$$ and $$k^C_2$$:
    - $$k^C_1 = L_k\left( \left( \textbf{x}^i\right) _{n}+\frac{1}{4}k_1+ \left( \frac{1}{4}- \frac{\sqrt{3}}{6}\right) k_2 \Delta t\right) $$;
    - $$k^C_2 = L_k\left( \left( \textbf{x}^i\right) _{n}+ \left( \frac{1}{4}+ \frac{\sqrt{3}}{6}\right) k_1 + \frac{1}{4}k_2\Delta t \right) $$;
- Updating the solution after the convergence of the implicit step is reached: $\left( x_k^i\right) _{n+1} = \left( x_k^i\right) _{n} + \frac{1}{2} \Delta t \left( k_1 + k_2 \right)$.

### Test 1: Dynamics on a single node with three species

As a validation, this numerical procedure is challenged against a well-known benchmark test involving three species on a single node. The payoff matrix chosen is RPS and reads:

3.3 $$\begin{aligned} RPS = \begin{pmatrix} 0 & 1 & -1 \\ -1 & 0 & 1 \\ 1 & -1 & 0 \\ \end{pmatrix}. \end{aligned}$$

**Fig. 1:**
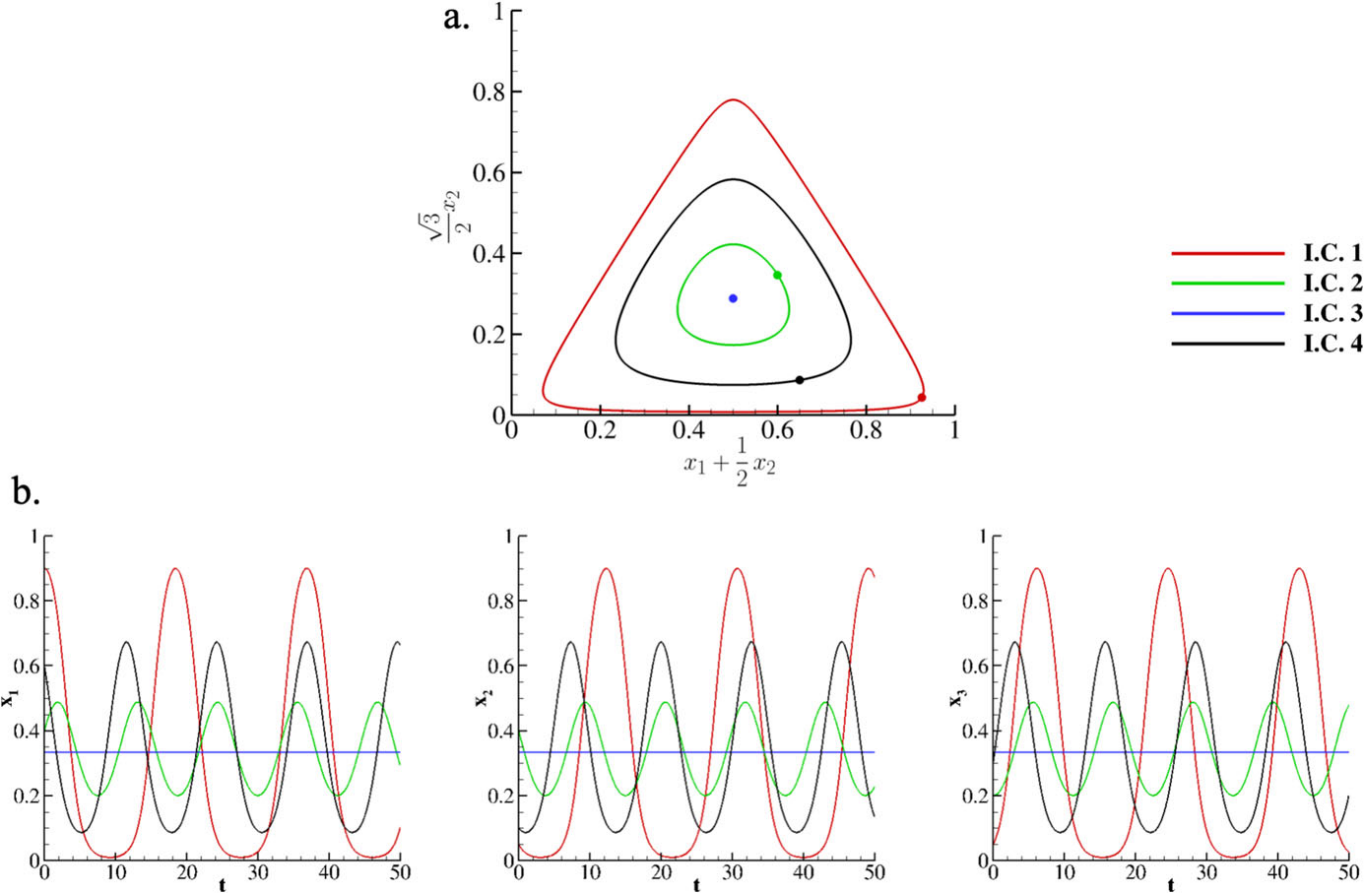
Distributions of three species for $A = RPS$ with four different initial conditions on the simplex coordinates **a.** and over time **b.**. In **a.**, the dots indicate initial conditions.

This payoff matrix is commonly associated with the game *rock, paper, and scissors*, and its skew-symmetric structure uniquely characterizes it; each win for one strategy corresponds to an equivalent loss for its opponent. This cyclic dominance (rock beats scissors, scissors beats paper, and paper beats rock) ensures that no single strategy is inherently superior, which is a critical aspect when analyzed under replicator dynamics. In this framework, the frequency of strategies in the population evolves according to their relative success: strategies performing better than average increase in prevalence, while those performing worse decrease. However, due to the zero-sum nature - if we sum the payoff matrix entries across all rows, the total is zero, and similarly, if we sum across all columns - the dynamics typically lead to perpetual oscillations around the unique mixed equilibrium point ($x_1=x_2=x_3=\frac{1}{3}$) rather than converging to an asymptotically stable state. This result vividly illustrates how diversity and cyclic competition can be maintained in evolutionary settings and gives, in turn, a suitable numerical experiment for validating the robustness of a scheme in terms of accuracy for large observation time. In this light, in Figure 1 the evolution of three species evolving according to *RPS* is provided for $\Delta t = 0.01$ and the following four different initial conditions:

$$\begin{aligned} {\textbf {I.C. 1:}} \qquad&(x_1(0),x_2(0),x_3(0)) = (0.9,0.05,0.05)\, , \\ {\textbf {I.C. 2:}} \qquad&(x_1(0),x_2(0),x_3(0)) = (0.4,0.4,0.2)\, , \\ {\textbf {I.C. 3:}} \qquad&(x_1(0),x_2(0),x_3(0)) = \left( \frac{1}{3},\frac{1}{3},\frac{1}{3}\right) \, , \\ {\textbf {I.C. 4:}} \qquad&(x_1(0),x_2(0),x_3(0)) = (0.6,0.1,0.3)\, . \end{aligned}$$

For representing the three trajectories $x_1(t)$, $x_2(t)$, and $x_3(t)$ on the simplex in a two-dimensional plot, a transformation from barycentric to Cartesian coordinates $\left( x_1+\frac{1}{2}x_2\,, \, \frac{\sqrt{3}}{2}x_2\right)$ is adopted; as such, the vertices of the standard simplex on $\mathbb {R}^3$ (except for the origin) are mapped as follows:

$$\begin{aligned} \text {- the vertex}&\, (1, 0, 0) \, \text { corresponds to } \left( 1, 0\right) , \\ \text {- the vertex}&\, (0, 1, 0) \, \text { corresponds to } \left( \frac{1}{2}, \frac{\sqrt{3}}{2}\right) , \\ \text {- the vertex}&\, (0, 0, 1) \, \text { corresponds to } \left( 0, 0\right) . \end{aligned}$$

**Fig. 2:**
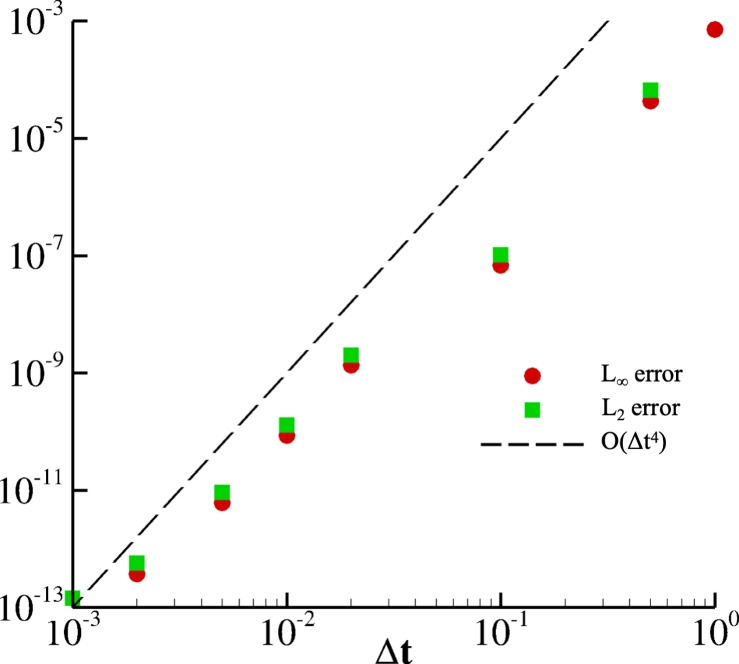
Decaying of L$_2$ and L$_\infty$ errors against decreasing $\Delta t$ for $A = RPS$. Data are reported in Table.1.

This transformation produces an equilateral triangle, which is particularly useful for visualizing the replicator dynamics and the trajectories within the simplex. As Figure 1.a demonstrates, the system remains in the asymptotically stable equilibrium point when choosing **I.C. 3**. On the contrary, the discussed cyclic dominance is obtained for all other initial conditions, thus returning loops on the simplex. For clarity, obtained trajectories are also provided over time in Figure 1.b and, in the same fashion, the different behaviors depending on the initial conditions. For measuring the accuracy of the adopted procedure, this numerical experiment is repeated for decreasing $\Delta t$ and **I.C.1**. In Table 1, we report $L_\infty$ and $L_2$ errors along with the relative experimental orders of convergence computed with respect to the solution for $\Delta t = 10^{-4}$ used as reference. Overall, the numerical procedure returns both errors decaying with O$(\Delta t^4)$, so a fourth-order accuracy is recovered (data are also graphically reported in Figure 2).

**Fig. 3:**
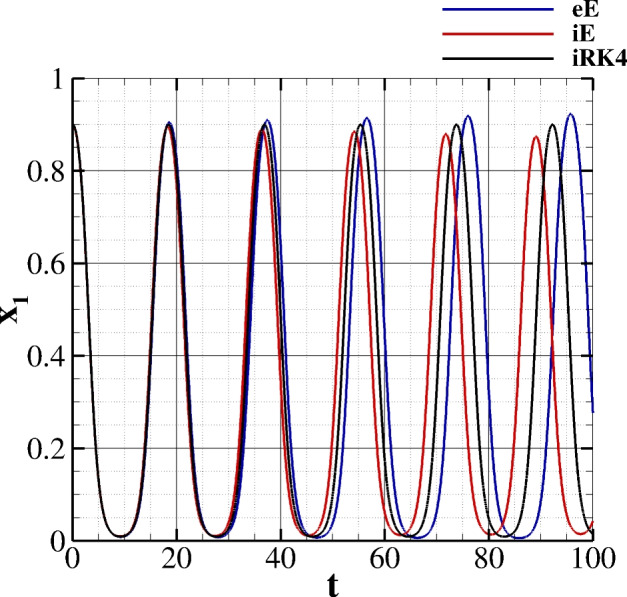
Comparison between the trajectory $x_1$ obtained for **I.C. 1** and $\Delta t = 0.01$ obtained with the present formulation (referenced as iRK4), the explicit Euler scheme (referenced as eE), and the implicit Euler scheme (referenced as iE).

A last comment regarding the adopted numerical scheme is in order. As known, there are numerous low-order symplectic schemes in the literature; however, in the context of the present paper, the accuracy of the model predictions for large observation times is essential, thus requiring a higher-order scheme. To demonstrate the authors’ point, the trajectory $x_1$ obtained for **I.C. 1** and $\Delta t = 0.01$ is compared to the ones approximated by explicit (eE) and implicit (iE) Euler scheme (see Figure 3). The approximation obtained with the explicit Euler method (blue line) shows significant amplitude growth over time, indicating numerical instability characteristic of explicit schemes when applied to stiff or oscillatory systems; the implicit Euler method (red line) exhibits pronounced numerical damping, with the oscillation amplitude decreasing substantially over the integration period. This behavior is typical of first-order implicit methods, which tend to be overly dissipative for oscillatory problems. The proposed method (black line) maintains remarkably stable amplitude throughout the simulation, suggesting superior conservation properties and numerical accuracy. The iRK4 trajectory closely preserves the system’s energy characteristics without the spurious amplitude growth or excessive damping experienced when adopting the two formulations of the Euler method. All three methods maintain similar oscillation frequencies, indicating that the time step is adequate for capturing the temporal dynamics, though the phase relationships may differ slightly due to the different truncation errors inherent in each scheme.

**Table 1:** L$_2$ and L$_\infty$ errors along with the relative experimental order of convergences (EOC$_\infty$ and EOC$_2$) obtained for decreasing $\Delta t$ on a single node for $A = RPS$.

$\Delta t$	L$_2$ error	EOC$_2$	L$_\infty$ error	EOC$_\infty$
$1.0 \times 10^{0}$	$7.119 \times 10^{-4}$	-	$1.0674 \times 10^{-3}$	-
$5.0 \times 10^{-1}$	$4.345 \times 10^{-5}$	3.18	$6.559 \times 10^{-5}$	3.34
$1.0 \times 10^{-1}$	$6.831 \times 10^{-8}$	3.79	$1.027 \times 10^{-7}$	3.80
$2.0 \times 10^{-2}$	$1.351 \times 10^{-9}$	3.96	$2.004 \times 10^{-9}$	3.96
$1.0 \times 10^{-2}$	$8.665 \times 10^{-11}$	3.99	$1.283 \times 10^{-10}$	3.99
$5.0 \times 10^{-3}$	$6.172 \times 10^{-12}$	3.96	$9.178 \times 10^{-12}$	3.96
$2.0 \times 10^{-3}$	$3.777 \times 10^{-13}$	3.29	$5.710 \times 10^{-13}$	3.30
$1.0 \times 10^{-3}$	$9.295 \times 10^{-14}$	1.11	$1.444 \times 10^{-13}$	1.11

### Test 2: Replicator dynamics on a network.

**Fig. 4:**
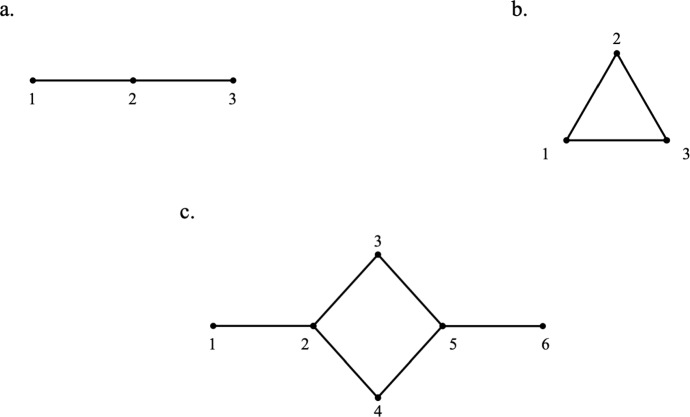
Network configurations with enlightened nodes indexing.

In the following, the two formulations for the transport term defined in Eq.s (2.1) and (2.2) are analyzed and characterized for two and three species evolving on a network. Three prototypical networks are considered: in-line (Figure 4.a), the triangle (Figure 4.b), and the roundabout (Figure 4.c). All provided computations are performed adopting $\Delta t = 0.01$. For the case of two species evolving on a network, the payoff matrix *HDG* for all nodes is chosen as a variant of the well-known *hawk-dove* game payoff matrix:

3.4 $$\begin{aligned} HDG = \begin{pmatrix} 2 & 3 \\ 4 & 1 \\ \end{pmatrix}\, . \end{aligned}$$

Such a matrix describes an evolutionary game modeling the conflict between two opposing strategies; i) the *dove* strategy, a peaceful strategy based on cooperation. When two *doves* interact, both receive a relatively good payoff, namely $HDG_{11} = 2$; ii) the *hawk* strategy, based on competition. When a *hawk* fights a *dove*, the first receives a high payoff, namely $HDG_{21} = 4$, while the *dove* (not entirely defeated) obtains a moderate payoff, namely $HDG_{12} = 3$. On the other hand, if two *hawks* meet, the cost of the conflict leads low payoff, namely $HDG_{22} = 1$. This structure, although not the standard formulation of the classic *hawk*-*dove* game (where typically $HDG_{12} = 0$, i.e., the *dove* receives zero when facing a *hawk*), represents a variant in which both species are rewarded with nonzero benefits in asymmetric encounters. This game has three equilibrium points: $(x_1,x_2) = (0,1)$, $(x_1,x_2) = \left( \frac{1}{2},\frac{1}{2} \right)$, and $(x_1,x_2) = (1,0)$ - being the mixed equilibrium point $\left( x_1,x_2) = (\frac{1}{2},\frac{1}{2} \right)$ the unique Nash equilibrium of the system. This matrix fulfills the hypotheses of Theorem 2.7. Regarding the case of the evolution of three species on a network, the discussed *rock-paper-scissors* game is selected. The rationale behind choosing those two games is that they are simple (while nontrivial) examples of games with an asymptotically stable mixed equilibrium point. In the following, the stability of such points will be challenged by the presence of the transport term in Eq. (2.3) for both systems. Specifically, for all networks, node 1 will be initialized with the asymptotically stable mixed equilibrium point, while the others will be initialized with pure equilibrium points. The ability of the model to perturb such pure equilibria will be discussed. Observe that, due to the simplex invariance shown in **Remark** 2.8, $x^i_k(t)\in S_n$ for all $t>0$ if $x^i_k(0)\in S_n$. However, due to the presence of the transport term, the invariance of the simplex support (classical property of the replicator dynamics on a single node (Sigmund and Hofbauer 1998; Weibull 1997) no longer holds. So that, a species $x^i_k$ that is null at a certain time $\tau \ge 0$ may be $x^i_k(t) > 0$ for $t>\tau$. Moreover, due to the attractiveness of the asymptotically stable equilibrium point, all trajectories are expected to converge to such points for arbitrarily large observation times.

#### Dynamic on a network with linear transport term.

Let the authors consider Eq. (2.3) with $\textbf{T}(\textbf{x}^i(\cdot )) = \textbf{T}^\nu (\textbf{x}^i(\cdot ))$ as defined in Eq. (2.1). In this section, for the three considered networks, the same payoff matrix will be provided for all nodes as well as for all *i* and $j\in \mathcal {I}$, $\nu _{ij}=\nu$. Note that, for all nodes belonging to the in-line and triangular networks $|c(i)| \le 2$ while for the roundabout network $|c(i)| \le 3$. Therefore, Eq. (2.7) is satisfied for the first two for $\nu < \frac{1}{2}$ and for the latter for $\nu < \frac{1}{3}$.

**Fig. 5:**
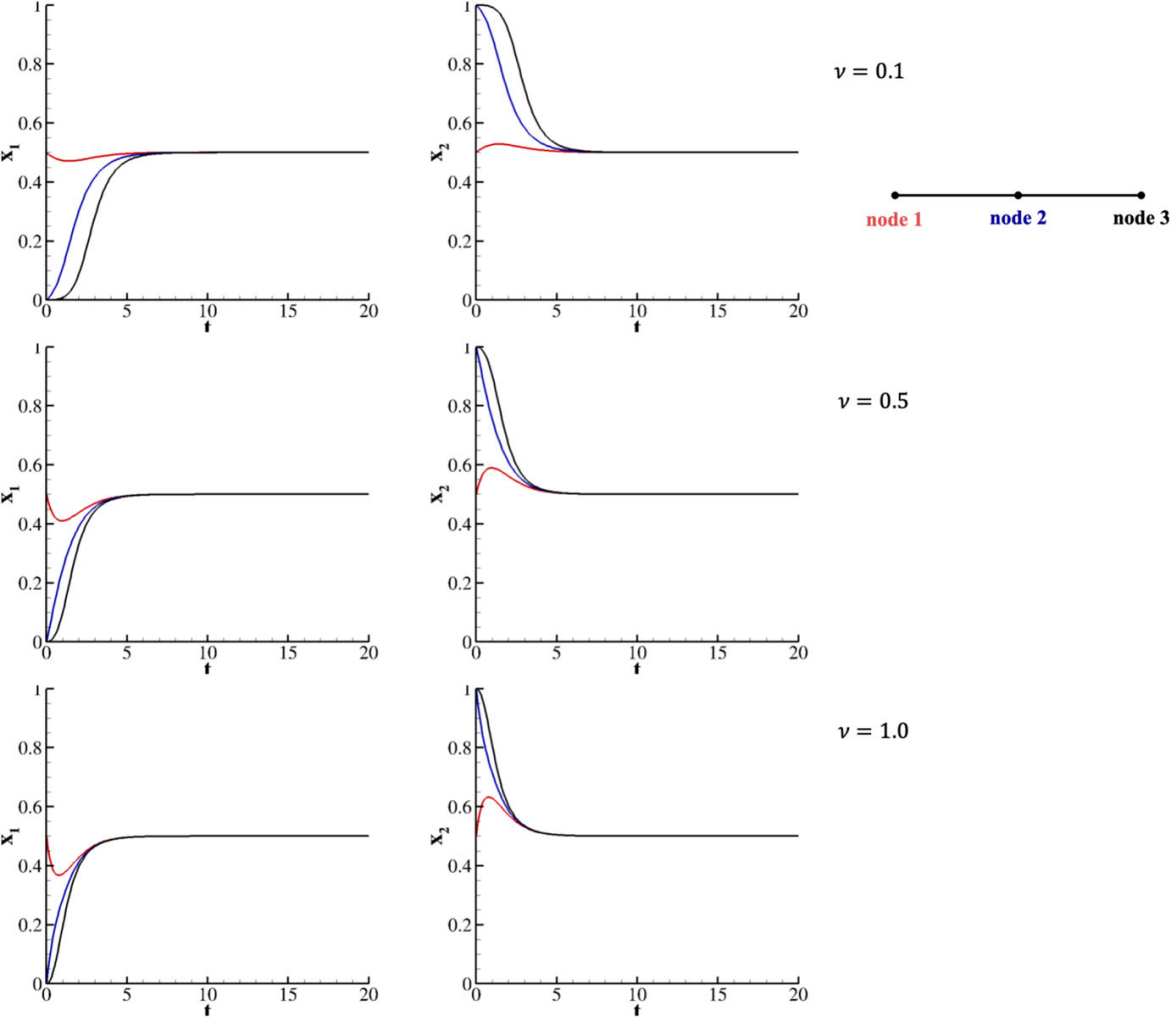
Two species evolving on an in-line network for $A=HDG$ and $x^1_1(0)=x^1_2(0)=\frac{1}{2}$; $x^2_1(0) = 0$, $x^2_2(0) = 1$; and $x^3_1(0) = 0$, $x^3_2(0) = 1$. Upper row corresponds to $\nu = 0.1$, middle row to $\nu = 0.5$, and lower row to $\nu = 1$.

Consider first the in-line network in Figure 4.a for the fitness matrix *HDG* and transport coefficient $\nu$. As discussed, this system is evolved by setting the following initial conditions: $x^1_1(0)=x^1_2(0)=\frac{1}{2}$; $x^2_1(0) = 0$, $x^2_2(0) = 1$; and $x^3_1(0) = 0$, $x^3_2(0) = 1$. Figure 5 documents the trajectories of $x_1$ and $x_2$ belonging to the three nodes. As expected, all nodes reach the asymptotic equilibrium point, but at different times depending on the transport term. Specifically, a larger value of $\nu$ results in a faster approach to equilibrium. For $\nu = 1$, equilibrium is reached at $\tau = 6.5$, whereas for $\nu = 0.5$ and $\nu = 0.1$, it occurs later, at $\tau = 6.8$ and $\tau = 8.2$, respectively. The presence of the transport term initially enables the null strategies $x^2_1$ and $x^3_1$ to reach the mixed equilibrium point. Notably, for $\nu = 0.1$, species in node 3 begin deviating from the pure equilibrium significantly later than those in node 2. This delay is due to the functional form of Eq. (2.1), which quantifies the difference in the $k$-th strategies between connected nodes. Initially, this difference is zero between species in nodes 2 and 3, whereas it is much larger between species in nodes 1 and 2.

The analysis of the error decaying for halving $\Delta t$ is provided in Table 2 for $\nu =$ 0.1, 0.5, and 1.0. As expected, the proposed numerical procedure maintains the fourth-order of accuracy when considering the linear transport term.

**Table 2:** L$_2$ error and experimental order of convergence obtained for halving $\Delta t$ for two species evolving on an in-line network for $\nu =$ 0.1, 0.5, and 1

	$\nu = 0.1$		$\nu = 0.5$		$\nu = 1.0$	
$\Delta t$	L$_2$ error	EOC$_2$	L$_2$ error	EOC$_2$	L$_2$ error	EOC$_2$
$1.00 \times 10^{-1}$	$4.001 \times 10^{-7}$	-	$8.755 \times 10^{-7}$	-	$6.391 \times 10^{-7}$	-
$5.00 \times 10^{-2}$	$2.506 \times 10^{-8}$	3.98	$5.261 \times 10^{-8}$	3.98	$3.845 \times 10^{-8}$	3.97
$2.50 \times 10^{-2}$	$1.567 \times 10^{-9}$	3.99	$3.224 \times 10^{-9}$	4.00	$2.357 \times 10^{-9}$	3.99
$1.25 \times 10^{-2}$	$9.798 \times 10^{-11}$	3.99	$1.919 \times 10^{-11}$	3.98	$1.457 \times 10^{-11}$	3.97
$6.25 \times 10^{-3}$	$2.125 \times 10^{-12}$	3.99	$2.471 \times 10^{-12}$	3.96	$2.063 \times 10^{-12}$	3.97

**Fig. 6:**
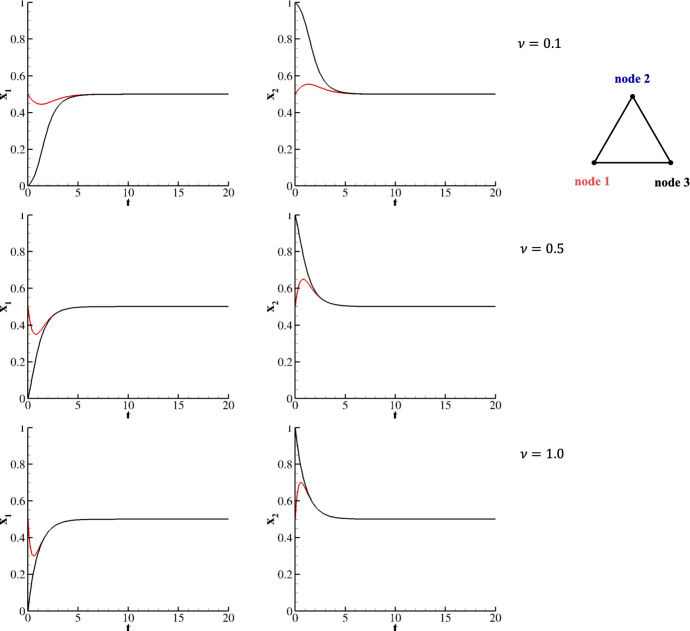
Two species evolving on a triangular network for $A=HDG$ and $x^1_1(0)=x^1_2(0)=\frac{1}{2}$; $x^2_1(0) = 0$, $x^2_2(0) = 1$; and $x^3_1(0) = 0$, $x^3_2(0) = 1$. Upper row corresponds to $\nu = 0.1$, middle row to $\nu = 0.5$, and lower row to $\nu = 1$.

In the same fashion as per the in-line network, we consider two species following the two possible strategies defined in HDG on a triangular network with initial conditions $x^1_1(0)=x^1_2(0)=\frac{1}{2}$; $x^2_1(0) = 0$, $x^2_2(0) = 1$; and $x^3_1(0) = 0$, $x^3_2(0) = 1$. They would achieve the mixed equilibrium point at $\tau =$ 6.8, 6.5, and 6.2 for $\nu =$ 0.1, 0.5, and 1, respectively (see Figure 6); indeed, since all nodes are connected, the trajectories of strategies belonging to nodes 2 and 3 completely overlap.

**Fig. 7:**
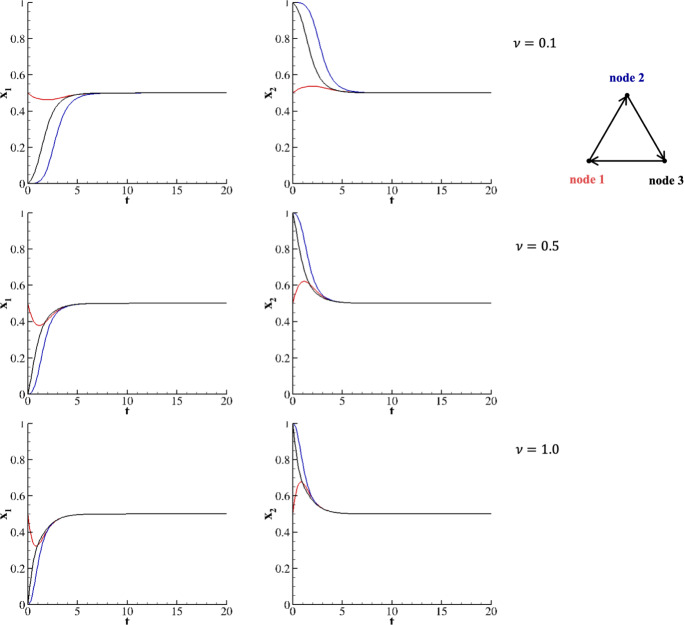
Two species evolving on a triangular oriented network for $A=HDG$ and $x^1_1(0)=x^1_2(0)=\frac{1}{2}$; $x^2_1(0) = 0$, $x^2_2(0) = 1$; and $x^3_1(0) = 0$, $x^3_2(0) = 1$. Upper row corresponds to $\nu = 0.1$, middle row to $\nu = 0.5$, and lower row to $\nu = 1$.

Interestingly, by providing an orientation to the triangular network (see Figure 7), the trajectories of species belonging to node 3 achieve the mixed equilibrium faster than those of node 2. This mechanism is due to the balance between analogous species in different nodes. Initially, $x^1_1$ decreases ($x^1_2$ increases), deviating from the mixed equilibrium state in favor of $x^2_1$ ($x^2_2$). However, at the same time, $x^3_1$ increases at the expense of $x^2_1$ ($x^3_2$ decreases in favor of $x^1_2$). So collectively, species in node 2 move away from the pure state more slowly than in node 3. As already observed for the in-line network, this mechanism is modulated by $\nu$.

**Fig. 8:**
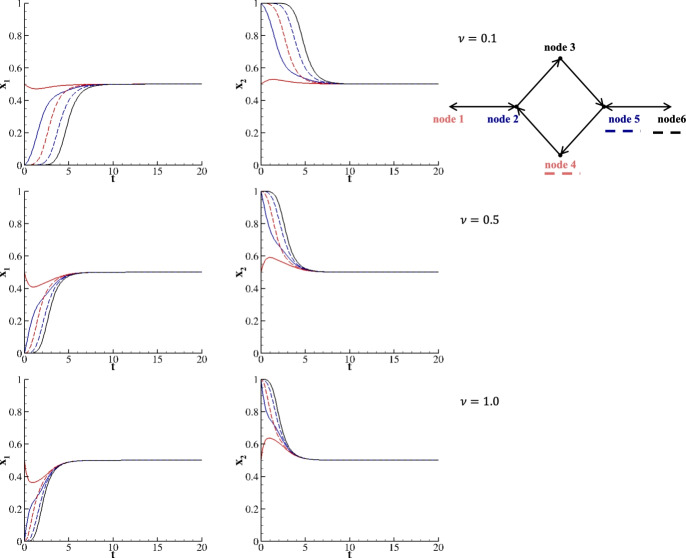
Two species evolving on an oriented roundabout network for $x^1_1(0)=x^1_2(0)=\frac{1}{2}$; $x^i_1(0) = 0$, $x^i_2(0) = 1$ for $i =$ 2, 3, 4, 5, and 6. Upper row corresponds to $\nu = 0.1$, middle row to $\nu = 0.5$, and lower row to $\nu = 1$.

Species evolving on the roundabout network show two different behaviors. This network can be decomposed into two in-line networks: node 1 - node 2 - node 3 - node 5 - node 6 (equivalently node 1 - node 2 - node 4 - node 5 - node 6). Therefore, by imparting identical initial conditions to nodes 3 and 4, it can be observed that the pathways or trajectories of the species associated with these nodes become indistinguishable. This observation is substantiated by the evidence presented in Figure 11. On the other side, the evolution of species in nodes 1, 2, 5, and 6 is analogous to that in the in-line network. As done for the triangular network, by providing an orientation, the trajectories belonging to nodes 3 and 4 differ, as demonstrated in Figure 8. Notably, the trajectories in nodes 3 and 6 now overlap, due to their identical initial conditions and relative position in the network. This suggests that species from seemingly diverse habitats may adopt similar strategies if, concerning their interactions with other environments, they find themselves in identical conditions at a given moment.

**Fig. 9:**
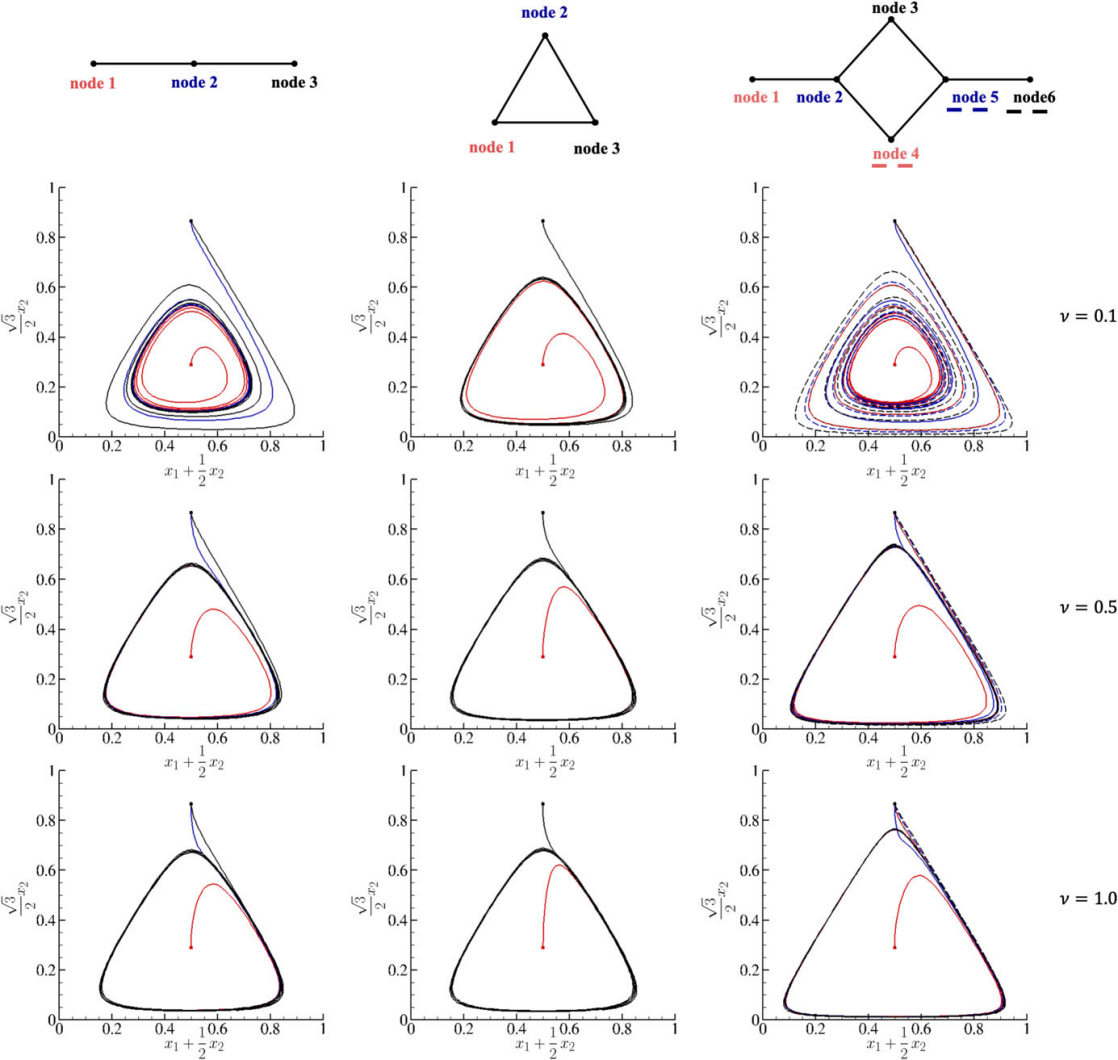
Plot of the trajectories of three species evolving on an inline (left plots), a triangular (central plots), and a roundabout network for $A=RPS$ on the simplex coordinates. With initial conditions $x_1(0)=x_2(0)=x_3(0)=\frac{1}{3}$ for the node 1 and $x_1(0) = 0$, $x_2(0) = 1$, and $x_3(0) = 0$ for all others nodes. Upper row corresponds to $\nu = 0.1$, middle row to $\nu = 0.5$, and lower row to $\nu = 1$.

Consider now three species evolving on such prototypical networks in which each node is ruled by $A = RPS$ discussed in Section 3. The latter admits a unique mixed equilibrium stable point $x_1=x_2=x_3=\frac{1}{3}$. As in the numerical experiments of the two species, node 1 will be initialized exactly at the mixed equilibrium point, while all other nodes will be initialized with the pure equilibria $x^i_1=0,\, x^i_2=1,\, x^i_3=0$ for $i \in \mathcal {I}-\{1\}$. Interestingly, also in this case, the system exerts the characteristic cyclic dominance of RPS. Figure 9 documents the obtained trajectories for the three networks and $\nu$ = 0.1, 0.5, and 1. For $\nu =0.1$, the attractiveness of the asymptotically stable equilibrium point is recovered for the in-line and roundabout networks; while for the triangular network, the trajectories almost immediately (for $t>20$) are completely synchronized across all nodes. On the other hand, for $\nu \ge 0.5$, the mixed equilibrium point seems to lose its attractiveness property, since all trajectories are found to synchronize on stable orbits. For clarity, all trajectories are also reported over time in Figures 12, 13, and  14.

#### Dynamic on a network with nonlinear transport term.

**Fig. 10:**
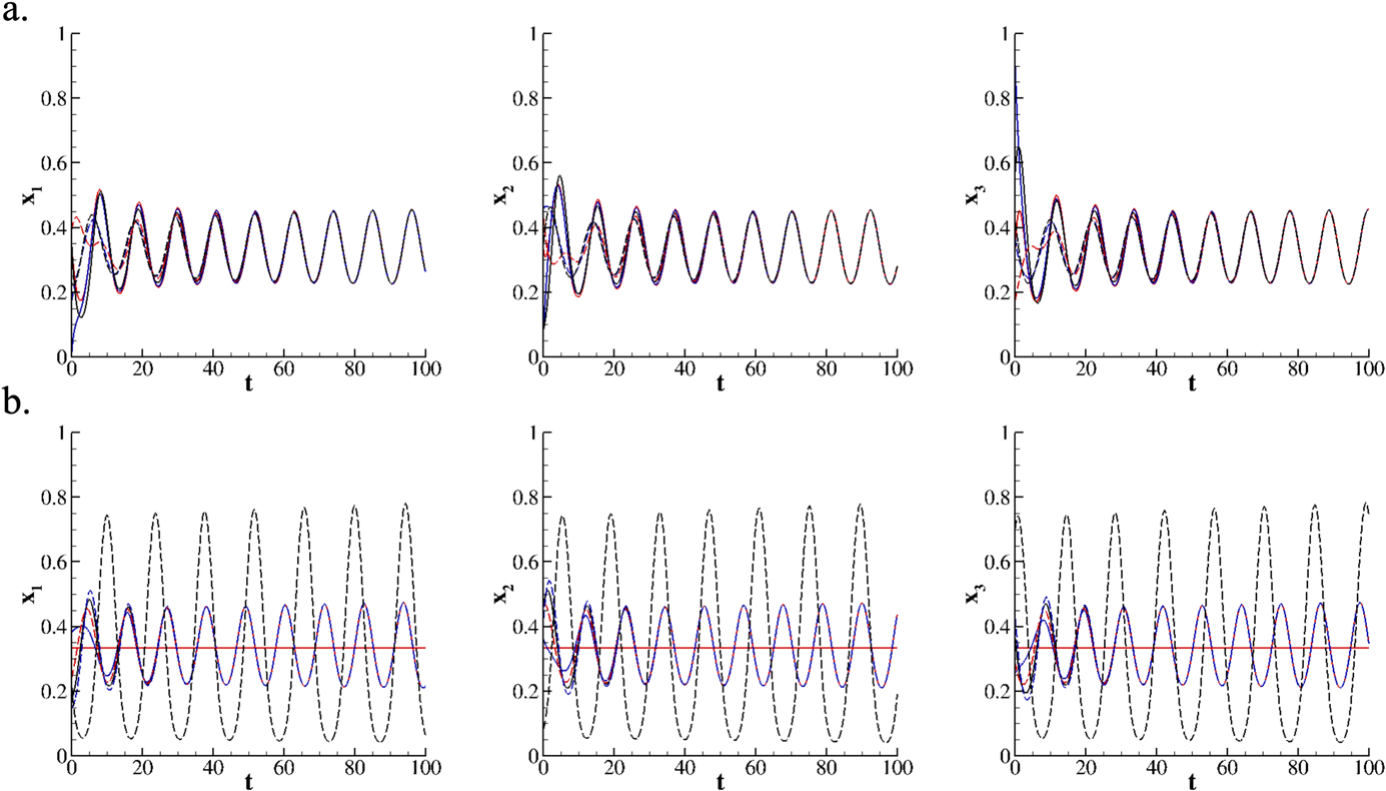
Three species evolving on a roundabout network for $A=RPS$, $p^{ij} = 2$ for all i and j in $\mathcal {I}$. Initial conditions are set as $x_1(0)=x_2(0)=x_3(0)=\frac{1}{3}$ for the node 1 and randomly extracted in [0, 1] for all others nodes. (**a.**) trajectories for $\tau ^1=\frac{1}{2}$ and $\tau ^2=1$. (**b.**) trajectories for $\tau ^1=5$ and $\tau ^2=1$. Red solid line is for node 1; blue solid line is for node 2; black solid line is for node 3; red dashed line is for node 4; blue dashed line is for node 5; black dashed line is for node 6.

Consider now three species evolving on a roundabout network following the nonlinear transport term defined in Eq.(2.2). For simplicity, $A=RPS$ is set on all nodes, and the survival rate is kept constant on the graph, $p^{ij} = p$ for all *i* and *j* in $\mathcal {I}$. The nodes are spaced such that $\tau ^{12}=\tau ^{56}=\tau ^1$ and $\tau ^{23}=\tau ^{35}=\tau ^{54}=\tau ^{42}=\tau ^2$. These assumptions represent three species originally from different environments, eager to find the optimal strategy for their flourishing and well-being through mutual interactions. Such interactions are regulated by the two traveling times - $\tau ^1$ and $\tau ^2$ - representing the cost of the interactions on the two in-line sub-networks (node 1 - node 2) and (node 5 - node 6), and within the quadrangle (node 2 - node 3 - node 5 - node 4), respectively. Moreover, it is assumed that those species may not experience completely safe journeys (due to predators, diseases, or any other ambient factor), so that a fifty-fifty survival rate is chosen, $p = 2$. The initial conditions are set as different for each node, specifically, $x_1(0)=x_2(0)=x_3(0)=\frac{1}{3}$ for node 1; while $\textbf{x}^i(0)$ are randomly extracted in the simplex $S^3$ for all other nodes. Figure 10.a documents the trajectories over time for $\tau ^1 = \frac{1}{2}$ and $\tau ^2=1$. Notably, the attractiveness of the asymptotically stable point $x_1=x_2=x_3=\frac{1}{3}$ is lost, and all species synchronize for $t > 75$. As observed in linear transport numerical experiments, due to the transport term, the state of node 1, initially set at the asymptotically stable point, is pushed onto a stable cyclic orbit together with all other nodes. Note that the ability of the proposed model to reproduce synchronization phenomena on networks is deeply relevant in the mathematical modeling of complex phenomena. An example is the synchronization of the fireflies flashing stimuli (Néda et al. 2000; Strogatz 2004). Synchronous fireflies represent a fascinating example of self-organization in nature: they possess the unique ability to coordinate their flashes of light through local interactions. Interestingly, regardless of the initial conditions chosen, this self-synchronization ability for the species on the network is modulated by the nodes’ spatial separation. In fact, by setting $\tau ^1 = 5$, we observe that node 1 and node 6 evolve as if they were almost unconnected to the network. Node 1 remains at the asymptotically stable point, as well as node 6 experiences the typical cyclic behavior described in Test 1 (see Figure 10.b). On the contrary, nodes belonging to the internal quadrangle form a sub-network into which all trajectories synchronize.

## Conclusive Remarks

This paper proposes a multiscale approach for the evolution of a marine ecosystem. The ecosystem is seen as a collection of interacting marine reserves mapped on a network. The evolution of species in local reserves is ruled by the replicator equation, while a transport function accounts for the transport at the network level. Two transport models are proposed: a linear transfer term based on the balance of a species between two nodes and a nonlinear term accounting for the time needed for a species to reach the selected node. For the linear case, the stability of the equilibrium states of the model is characterized, and it is proved that evolutionarily stable states are asymptotically stable if the transfer velocity is modulated by the maximum degree of the network. A fourth-order P-(EC)k formulation of the Gauss-Legendre Runge Kutta scheme is proposed for integrating the model on three prototypical networks. This numerical procedure is challenged against suitable numerical experiments involving two and three species on such networks for large observation times with both linear and nonlinear transport functions. The computational procedure was validated in terms of its robustness and accuracy, returning an experimental fourth order of convergence. The model and the computational approach were shown to have unique abilities in efficiently describing dynamics, long-term behavior, stability, and evolution of complex ecosystems. Finally, while the proposed model is designed to capture the dynamics of marine ecosystems, a qualitative validation against empirical observations remains to be undertaken. Future work will focus on establishing collaborations with biologists and related experts to access relevant datasets and advance the development of realistic, data-driven applications.

## Figures and Tables

**Fig. 11:**
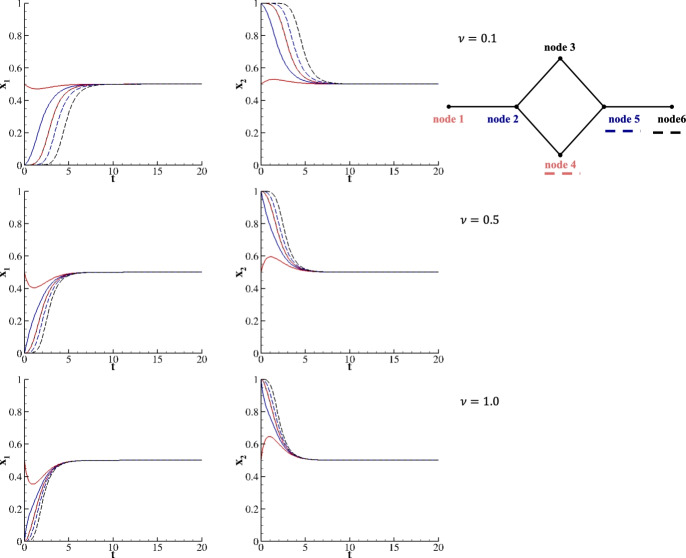
Two species evolving on a roundabout network for $x^1_1(0)=x^1_2(0)=\frac{1}{2}$; $x^i_1(0) = 0$, $x^i_2(0) = 1$ for $i =$ 2, 3, 4, 5, and 6. Upper row corresponds to $\nu = 0.1$, middle row to $\nu = 0.5$, and lower row to $\nu = 1$.

**Fig. 12:**
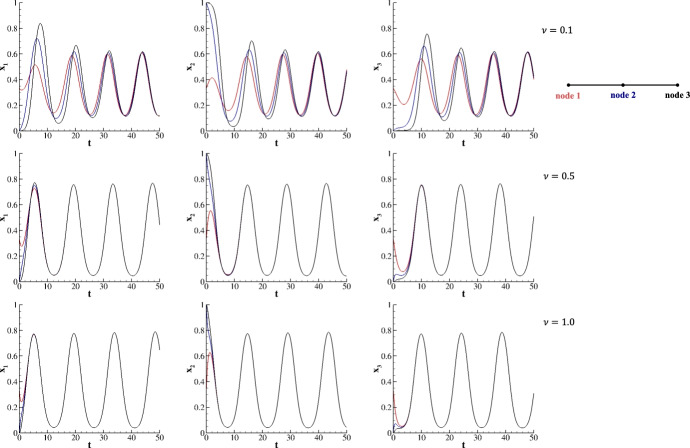
Three species evolving on an inline network for $A=RPS$ and $x^1_1(0)=x^1_2(0)=x^1_3(0)=\frac{1}{3}$; $x^i_1(0) = 0$, $x^i_2(0) = 1$, and $x^i_3(0) = 0$ for $i =$ 2 and 3. Upper row corresponds to $\nu = 0.1$, middle row to $\nu = 0.5$, and lower row to $\nu = 1$.

**Fig. 13:**
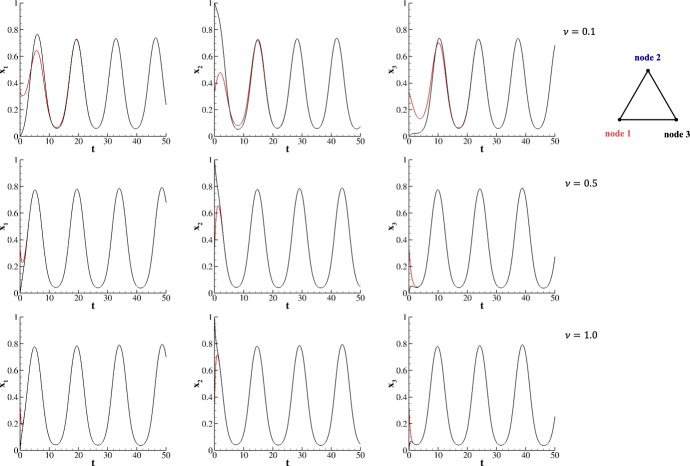
"Three species evolving on a triangular network for $A=RPS$ and $x^1_1(0)=x^1_2(0)=x^1_3(0)=\frac{1}{3}$; $x^i_1(0) = 0$, $x^i_2(0) = 1$, and $x^i_3(0) = 0$ for $i =$ 2 and 3. Upper row corresponds to $\nu = 0.1$, middle row to $\nu = 0.5$, and lower row to $\nu = 1$.

**Fig. 14:**
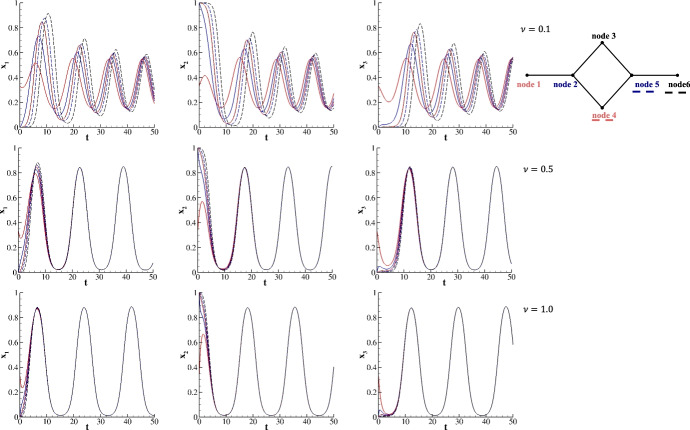
Three species evolving on a roundabout network for $A=RPS$ and $x^1_1(0)=x^1_2(0)=x^1_3(0)=\frac{1}{3}$; $x^i_1(0) = 0$, $x^i_2(0) = 1$, and $x^i_3(0) = 0$ for $i =$ 2, 3, 4, 5, and 6. Upper row corresponds to $\nu = 0.1$, middle row to $\nu = 0.5$, and lower row to $\nu = 1$.
